# Real time PCR for the rapid identification and drug susceptibility of Mycobacteria present in Bronchial washings

**DOI:** 10.1186/s12879-016-1943-y

**Published:** 2016-10-26

**Authors:** Thilini Piushani Keerthirathne, Dhammika Nayoma Magana-Arachchi, Dushantha Madegedara, Suneth Sithumini Sooriyapathirana

**Affiliations:** 1 National Institute of Fundamental Studies, Kandy, Sri Lanka; 2 Respiratory Disease Treatment Unit &Teaching Hospital, Kandy, Sri Lanka; 3 Department of Molecular Biology and Biotechnology, University of Peradeniya, Peradeniya, Sri Lanka

**Keywords:** Multiplex real-time PCR, MCAG, MAC, NTM, SYBR green

## Abstract

**Background:**

Mycobacteria have a spectrum of virulence and different susceptibilities to antibiotics. Distinguishing mycobacterial species is vital as patients with non-tuberculous mycobacterial (NTM) infections present clinical features that are similar to those of patients with tuberculosis. Thus, rapid differentiation of *Mycobacterium tuberculosis* complex from NTM is critical to administer appropriate treatment. Hence the aim of the study was to rapid identification of mycobacterial species present in bronchial washings using multiplex real time Polymerase Chain Reaction (PCR) and to determine the drug susceptibility in identified mycobacterial species.

**Methods:**

Sputum smear negative bronchoscopy specimens (*n* = 150) were collected for a period of one year, from patients attending the General Hospital Kandy, Sri Lanka. The specimens were processed with modified Petroff’s method and were cultured on Löwenstein– Jensen medium. DNA, extracted from the mycobacterial isolates were subjected to a SYBR green mediated real time multiplex, PCR assay with primers specific for the *M. tuberculosis* complex, *M. avium* complex, *M. chelonae-M.abscessus* group and *M. fortuitum* group. DNA sequencing was performed for the species confirmation, by targeting the 16S rRNA gene and the drug susceptibility testing was performed for the molecularly identified isolates of *M. tuberculosis* and NTM.

**Results:**

The optimized SYBR Green mediated multiplex real-time PCR assay was able to identify the presence of genus *Mycobacterium* in 25 out of 26 AFB positive isolates, two *M. tuberculosis* complex, three *M. avium* complex and two isolates belonging to *M. chelonae-M. abscessus* group. DNA sequencing confirmed the presence of *M. tuberculosis, M. chelonae-M. abscessus, M. intracellulare, M. avium*, *Rhodococcus* sp. and *M. celatum*. Remaining isolates were identified as *Mycobacterium* sp. All the NTM isolates were sensitive to amikacin and seven were resistant to ciproflaxacin. Twenty two of the NTM isolates and the isolate *Rhodococcus* was resistant to clarithromycin. The two isolates of *M. tuberculosis* were sensitive to all first line anti tuberculosis drugs.

**Conclusion:**

The optimized SYBR Green mediated multiplex real time PCR assay could be an effective tool for the rapid differentiation of pathogenic *M. tuberculosis* complex from the opportunistic nontuberculous mycobacteria and also it confirmed the presence of NTM in 15.3 % of the study population.

## Background

Tuberculosis (TB) is an infectious disease caused by strains of *Mycobacterium tuberculosis* complex (MTC) which is currently one of the leading causes of death in the world [1]. Alternatively, pulmonary infections caused by non-tuberculous mycobacteria (NTM), i.e. *Mycobacterium* species that are not members of MTC are also in the rise [2]. The increasing prevalence of the multi-drug resistant (MDR) TB strains are an added intimidation to the global TB burden, due to their resistance to the most effective first line anti-tuberculous drugs rifampin (RIF) and isoniazid (INH) [3]. The incidence of demises due to NTM related pulmonary infections have increased globally [4] and the rapid increase in the incidence of NTM disease in AIDS patients have also been recorded. Mycobacteria can be broadly classified as rapid growers (<7 days) and slow growers (>7 days) [5]. Fast growers commonly have two identical copies of the 16S rRNA gene, whereas slow growers have only one single copy [6]. When a case of mycobacterial infection is detected, both the clinical and public health management depend critically on whether the causative agent is a MTC or a NTM to prevent clinical misleading during therapeutic actions, as patients with NTM infections show clinical signs that are similar to those of patients with TB [7]. Mycobacteria have a spectrum of virulence and different susceptibilities to antibiotics and it is believed that altered target proteins produced due to specific gene alterations such as mutations, insertions or deletions are influencing the degree of susceptibility to the drug [8]. Though it is anticipated that the patient’s symptoms are mainly due to *M. tuberculosis* (MTB), the role of co-infecting NTM species in the pathogenesis of the pulmonary disease is still unclear. The presence of NTM in the patients with chronic TB has a substantial impact on clinical management. The treatment of diseases with MTC and NTM are different because many of the first and second line anti-tuberculous drugs are ineffective against many NTM [2]. Incorrect diagnosis of pulmonary diseases, i.e. if priority is given only for MTC and avoiding the presence of NTM, will lead to inappropriate treatments and the patients will not respond to conventional therapy [9].

As conventional detection methods such as, biochemical tests consume time [2], currently DNA amplification using Polymerase Chain Reaction (PCR) has allowed great progress in the rapid and accurate diagnosis of mycobacterial infections. Innovations in real –time PCR technology have eased and advanced the PCR methodology extensively. Application of SYBR green mediated real time PCR assay in clinical microbiology had improved the diagnostics due to the increased specificity and the ability to detect two or more organisms in a single reaction [10]. Rapid availability of the results is an added advantage of SYBR green mediated real time PCR assay over conventional PCR. Rapid identification will assist in avoiding unnecessary drug exposure and could aid in reducing the mortality and morbidity.

Incidence of TB in Sri Lanka was 66 in 2013. Human immunodeficiency virus (HIV) testing of all TB patients was made mandatory in the country since 2013. In 2013, 4646 TB patients were screened for HIV and out of these patients, six were found positive. In addition, there were nine patients with known HIV status at the time of diagnosis of TB, contributing to a total of 15 patients with HIV/ TB co-infection in 2013 [11]. In Sri Lanka, highest priority is given for the definitive diagnosis of TB and due to the limited records [12] about the NTM in clinical specimens, no testing is carried out for definitive diagnosis of NTM infections. Therefore any person with symptoms suggestive of TB, particularly cough for more than three weeks are being investigated only for TB and similar to other developing countries, routine diagnosis of TB in Sri Lanka is done with the sputum microscopy which could lead to the misdiagnosis of the pulmonary disease.

In our previous study we demonstrated the usefulness of bronchial washings as the preferred specimen in diagnosis of NTM lung disease by culture as well as from molecular techniques and about 13 to 14 % of the study population had NTM in their bronchial washings [12]. Hence in this study we assessed the utility of SYBR green mediated multiplex, real time PCR for the rapid identification of *Mycobacterium* species present in bronchial washings and also determined the drug sensitivity patterns of the identified mycobacterial isolates.

## Methods

### Study setting, population and ethics

Sputum smear negative bronchoscopy specimens were collected from patients (*n* = 150) attending the General Hospital Kandy, Sri Lanka from January 2014 to March 2015 who had pulmonary symptoms, nodular or cavitary opacities on chest radiograph, or an HRCT scan that showed multifocal bronchiectasis with multiple small nodules. Approval for the study was obtained from the Ethical Review Committee of the Postgraduate Institute of Science, University of Peradeniya, Sri Lanka. The bronchoalvelar lavage (BAL) specimens were collected as a part of routine investigations and a portion of the specimens were used for culturing. Written informed consent was obtained from each patient before bronchoscope and collecting the BAL specimens.

## Laboratory analysis

### Sample processing, culture and DNA extraction

The samples were liquefied and decontaminated with 4 % NaOH according to the modified Petroff’s method [13]. The decontaminated specimens were inoculated onto Löwenstein–Jensen media (L-J media), LJ media containing Thiophene – 2 carboxylic acid hydrazide (TCH) and p-nitrobenzioc acid (PNB) and cultures were observed for 8 to 12 weeks incubating at 37 °C and at 28 °C in light and dark conditions. Solid medium slants were considered positive when visible colonies grew. The colonies were further confirmed as mycobacteria by the Ziehl–Neelsen stain. All cultures that showed mycobacterial growth were subjected to further analysis. Genomic DNA was extracted from the mycobacterial isolates according to the standard CTAB (N-Cetyl-N, N, N-trimethyl ammonium bromide) method [14].

### Real - time multiplex PCR analysis

PCR primers targeting the internal transcribed spacers (ITSs) of MTC and *M. chelonae-M. abscessus* group (MCAG), the 16S rRNA genes of *M. avium* complex (MAC), *M. fortuitum* group (MFG) and the region of the 16SrRNA gene common to all the members of genus *Mycobacterium* which were described previously [10] were used for the optimizing of real -time multiplex, PCR assays (Table 1). The assays were performed on a real time PCR Instrument System (Rotor-GeneQ) and the DNA amplifications were monitored by the measurement of the SYBR Green fluorescence. The real time multiplex PCR assay was conducted in two separate reactions where primers specific for MTC and MAC were in reaction I and primers specific for MCAG and MFG were in reaction II which helped in identifying slow and rapid growers respectively. Primers targeting genus *Mycobacterium* were included in both the reactions (Table 1). Each reaction was carried out in a 25 μl volume which contained 2.0 μl of 25 mM MgCl_2,_ 0.25 μl of 5 u /μl Taq polymerase, 5.0 μl of 5X PCR Buffer, 2.5 μl of 1 mM dNTP mix, 1.0 μl of each primer (10 μm), 1.25 μl of 2X SYBR green and 50 ng of the extracted mycobacterial DNA. The PCR amplification process was initiated by ramping the temperature at 95 °C for 5 min followed by 40 cycles of the amplification process (95 °C for 15 s, 60 °C for 30s and 72 °C for 30s). Subsequent to the cycling process, melting curves were generated by inclining the temperature from 60 °C to 95 °C at 0.2 °C/s.

**Table 1:** Primers used for SYBR Green mediated, multiplex real-time PCR assay [10]

Organism	Target region	Primer sequence (5’–3’)	Reaction
*Mycobacterium avium*	16S	F:CCTCAAGACGCATGTCTTC	I
		R: ACCTACCGTCAATCCGAGAA	
*Mycobacterium intracelulare*	16S	F:GACCTTTAGRCGCATGTCTTT	I
		R: ACCTACCGTCAATCCGAGAA	
*Mycobacterium tuberculosis* Complex	ITS	F: GCGAGAGCCGGGTGCATG	I
		R: AACAGTGTGTTGGTGGCCAA	
AFB genus	16S	F: CCGCAAGRCTAAAACTCAAA	I/II
		R: TGCACACAGGCCACAAGGGA	
MCAG	ITS	F: TAAGGAGCACCATTTCCCAG	II
		R: CGACGTTTTGCCGACTACC	
MFG	16S	F: CCACGCGCTTCATGGTGT	II
		F: CCGCGCTCTTCATGGGGT	
		F: ACCACGCATTTCATGGTGT	
		R:ACTTGCGCTTCGTCCCTAT	

### DNA sequencing for species confirmation

For the species confirmation, the extracted DNA of the mycobacterial isolates were subjected to amplification of the 16S rRNA gene with the use of universal primers, F: 5’ TGGAGAGTTTGATCCTGGCTCAG 3’ and R: 5’ AAGGAGGTGATCCATC 3’ [10]. The amplified DNA was visualized from gel documentation system (SYNGENE) after the electrophoresis in 1.5 % agarose and ethidium bromide staining. 1kbp DNA marker was used to identify correct fragment of 1.5kbp. Amplified DNA fragments were purified using gel extraction kit (Promega) and were commercially sequenced from Macrogen Inc., South Korea using ABI 3730XL sequencers. The sequence data obtained were analysed using the software program BioEdit 7.0.9. The results were compared with the sequences from the GenBank database and the obtained sequences were deposited in the GeneBank database.

### Drug susceptibility analysis

The drug susceptibility testing for both MTC (*n* = 2) and NTM (*n* = 22) were carried out in duplicate with freshly grown cultures using the methods of agar proportion and the disk diffusion [15]. Susceptibility to isoniazid (INH) (0.2 μg/ml, 1.0 μg/ml), rifampin (RIF) (0.2 μg/ml), streptomycin (2 μg/ml, 10 μg/ml), ethambutol (5.0 μg/ml,10 μg/ml) and pyrazinamide (25 μg/ml) were determined with the agar proportion method [16] performed on Middle Brook 7H11 (MB7H11) agar media enriched with the oleic albumin dextrose catalase (OADC) supplement. The standard strain of H_37_Rv was used as the positive control. MB7H11 agar plates of 4–5 mm thickness, enriched with the OADC supplement was used for the susceptibility testing of the drugs amikacin (30 μg), ciprofloxacin (5 μg) and clarithromycin (15 μg) incorporated disks, purchased commercially from Hardy Diagnostics, Santa Maria, CA. At the same time, the viability of the disks were tested using the control strains of *Escherichia coli* ATCC 25922, *Pseudomonas aeruginosa* ATCC 27853 and *Staphylococcus aureus* ATCC 25923 on Mueller-Hinton Agar medium. Susceptibility patterns of these organisms were compared as stated in Clinical and Laboratory Standards Institute standard operating procedures [14].

## Results

### Participants and the culture characteristics

Of the 150 patients included in the study, 93 were males. The men: women ratio was 1.6:1. The age range was 12 to 83 years. 32 % of the males were above the age of 60 years while in females only 11 % were above the age of 60 years. But in the age group of 15–60 years, female percentage was higher (32 %) in comparison to the male patients (24 %). Of the 150 specimens, 52 had a growth within eight weeks of incubation. Overall, twenty three of 150 patients (15.3 %) were positive for any *Mycobacterium* species by culture (by confirmation of AFB). Out of these 23 patients, 14 were females (60.8 %) and MTB was identified in two of them. When mycobacterial culture was considered as the gold standard there were 26 isolates (17.3 %) from 23 patients belonging to the *Mycobacterium* genus and among theses 23 (15.3 %) were NTM. All the mycobacterial cultures were rapid growers except for two isolates. Mixed growths were observed from the two bronchial wash specimens. The sample KCC2 yielded three different isolates (02RC, 02YC and 02WC) and the sample KCC 6 yielded two isolates (06 RC and 06PL) and all these culture isolates were confirmed as AFB positive by ZN staining.

### Analysis of SYBR green mediated real time multiplex PCR assay

**Fig. 1:**
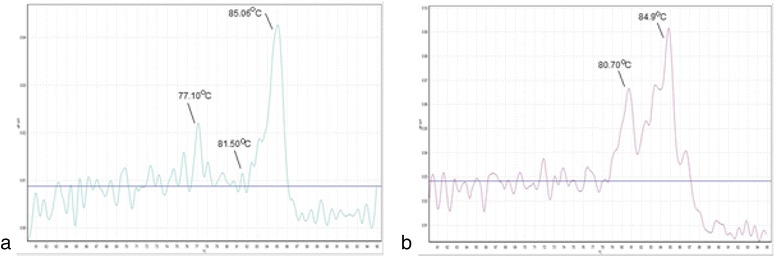
**a** SYBR green mediated real-time PCR melting curve, Tm 77.10 °C, 81.50 °C and 85.06 °C indicating the presence of MTC, genus *Mycobacterium* and MAC following Reaction I. **b** SYBR green mediated real-time PCR melting curve, Tm 80.70 °C and 84.90 °C indicating the presence of genus *Mycobacterium* and MFG following Reaction II

Initial assessment of the PCR primers for the specificity and the sensitivity of the genus *Mycobacterium* and MTC was done in a single–plex real time PCR with a standard H37Rv strain, an isolate of *M. intracellulare* and *M. phocaicum*, and a collection of previously identified clinical isolates of MTB. The presence of the amplicon was detected by the fluorescent dye SYBR green and the species specificities were defined by the analysis of melting curves. In Reaction 1, a DNA template from MTB produced a peak with a *T*_*m*_ ~ 77 °C, which corresponded to MTC, and a peak with a *T*_*m*_ ~ 80–83 °C which corresponded to the genus *Mycobacterium* respectively. Similarly DNA from MAC produced a peak with a *T*_*m*_ ~ 85 °C, which corresponded to MAC (Fig. 1a). In Reaction II, a DNA template from MCAG produced a peak with a *T*_*m*_ ~ 77 °C, which corresponded to MCAG, and a peak with a *T*_*m*_ ~ 80–83 °C corresponded to the genus *Mycobacterium* respectively. Similarly in Reaction II, DNA from *Mycobacterium* spp. produced peaks with *T*_*m*_ ~ 84–89 °C, which corresponded to MFG and other mycobacteria and a peak with a *T*_*m*_ ~ 80–83 °C which corresponded to the genus *Mycobacterium* (Fig. 1b). When the mycobacterial DNA of the study population were subjected to Reaction I of the SYBR green mediated real time multiplex PCR assay, it confirmed the presence of *Mycobacterium* genus in 25 of the 26 AFB positive isolates, in two MTC (*n* = 2) isolates and three MAC (*n* = 3) isolates. Evaluation of the analysis of Reaction II also confirmed the presence of genus *Mycobacterium* in tested mycobacterial isolates and also the presence of MCAG in two isolates (*n* = 2).

### Analysis of DNA sequencing

DNA sequencing confirmed the presence of a *Rhodococcus* sp. (*n* = 1), a *Mycobacterium celatum* (*n* = 1), *M. chelonae*-*abscessus* complex (*n* = 3), two isolates were identified as *M. intracellulare* and the other isolate as *M. avium*. The isolates identified as MTC were confirmed as MTB and the remaining isolates could be only identified as belonging to genus *Mycobacterium*.

### Analysis of drug susceptibility testing

All the NTM isolates were sensitive to amikacin and seven were resistant to ciproflaxacin. Except for the two isolates of *M. chelonae*-*abscessus* complex and a single isolate of *M. intracellulare*, all the other isolates of NTM were resistant to clarithromycin (Table 2). None of the isolates were resistant to all three tested drugs. The two isolates of *M. tuberculosis* were sensitive to all first line anti tuberculosis drugs.

**Table 2:** Effect of clarithromycin, amikacin and ciprofloxacin on the identified NTM isolates

SI number	Isolate	Organism	Drug		
			Clarithromycin	Amikacin	Ciprofloxacin
1.	KCC 142	*Mycobacterium* sp. (KU198858)	R	S	S
2.	KCC 06 PL	*Mycobacterium chelonae*-*abscessus complex* (KU179046)	S	S	R
3.	KCC 124	*Mycobacterium chelonae*-*abscessus complex* (KU195328)	S	S	R
4.	KCC 06 RC	*Mycobacterium chelonae*-*abscessus complex* (KU195330)	R	S	R
5.	KCC119	*Mycobacterium celatum* (KU179047)	S	S	R
6.	KCC 02 YC	*Rhodococcus* sp. (KU198859)	R	S	S
7.	KCC 72	*Mycobacterium* sp.(KU195326)	R	S	R
8.	KCC 82	*Mycobacterium* sp.(KU195322)	R	S	S
9.	KCC 131	*Mycobacterium avium complex*(KU198858)	R	S	S
10.	KCC 94	*Mycobacterium* sp.(KU377301)	R	S	S
11.	KCC 98	*Mycobacterium* sp.(KU195324)	R	S	S
12.	KCC 90	*Mycobacterium intracellulare* (KU195323)	S	S	S
13.	KCC 101	*Mycobacterium* sp.(KU195327)	R	S	S
14.	KCC 78	*Mycobacterium* sp.(KU195320)	R	S	R
15.	KCC 83	*Mycobacterium* sp. (KU377300)	R	S	S
16.	KCC 129	*Mycobacterium* sp.(KU195325)	R	S	S
17.	KCC 76	*Mycobacterium intracellulare* (KU195319)	R	S	S
18.	KCC 81	*Mycobacterium* sp.(KU195321)	R	S	S
19.	KCC 97	*Mycobacterium* sp. (KU377299)	R	S	S
20.	KCC 59	*Mycobacterium* sp. (KU195318)	R	S	S
21.	KCC 132	*Mycobacterium* sp.	R	S	S
22.	KCC 73	*Mycobacterium* sp.	R	S	R

R = resistant S = sensitive

## Discussion

Conclusive diagnosis of a mycobacterial infection commonly involves isolation and identification of the infecting organism in the laboratory by culture, even though, the preliminary diagnosis is frequently based on clinical data [17]. But even when cultures are detected positive and the presence of mycobacteria were confirmed by ZN staining it is not yet possible to confirm it as a MTC or a NTM [18]. To address this issue we have optimized a molecular assay capable of identifying MTC and NTM when applied to positive cultures on LJ; further identification of species is achieved by the sequencing of 16SrDNA sequencing. In this study, 25 clinical isolates were identified in terms of species, group or to complex level with the SYBR green mediated real time multiplex, PCR assay, keeping 16S rRNA gene sequencing as the gold standard. The PCR assay indicated the presence of two MTB isolates and 23 NTM isolates in the collected bronchial washings. However 16S rRNA gene sequencing confirmed that one of the isolate was belonging to the genus *Rhodococcus* which was positive in ZN stain. 16S amplicons from two NTM positive cultures failed to yield DNA sequence data and as such further identification of these two isolates could not be made. When mycobacterial culture was considered as the gold standard there were 26 isolates (17.3 %) belonging to the *Mycobacterium* genus and among theses 23 (15.3 %) were NTM.

In the past two decades, nucleic acid amplification-based techniques such as PCR have become accessible to the clinical mycobacteriology laboratory. Sequence based methods are more rapid and accurate than the conventional identification methods. Most PCR protocols have concentrated on the detection of pathogenic MTB as they claim more human lives than any other bacteria. Both in house and commercial PCRs have been widely evaluated [1, 2, 10, 19]. Real-time PCR techniques, involving fluorescent dyes or fluorophores with a spectrofluorometric thermal cycler, have been used to develop a number of rapid and sensitive assays for identification of bacteria and viruses [20]. Several fluorescence formats are available for the detection of amplified DNA of MTB. Most of these assays have used SYBR Green, a double-stranded DNA (dsDNA) binding dye that fluoresces when bound to dsDNA [21]. Fluorimeter-based analysis also has provided a rapid and sensitive method for identification of PCR products [21]. However, even species specific real-time PCR, have their limitations since it only recognises selected species of interest.

The molecular identification procedure which was elucidated in this study intended to identify rapid and slow growing mycobacteria in two separate reactions. Hence this assay was able to differentiate the pathogenic mycobacterial presence with the use of these two parallel reactions. Including the *Mycobacterium* genus specific primer set in both reactions was to use it as a positive internal control which facilitated the identification of the presence of various *Mycobacterium* species, though the absence of the targeted organisms. A third of the world population is thought to be infected with TB [21]. The developing countries including Sri Lanka, are still suffering from TB, mainly due to poverty (lack of healthy living conditions) and satisfactory medical care [22]. There were two *M. tuberculosis* strains identified during the study, both the strains were sensitive for the first line anti TB drugs indicating that those were treatable tuberculosis cases. DNA from the identified *M. tuberculosis* isolates were amplified, sequenced and deposited on to the GenBank data base with the accession numbers KU179045 and KU195329.

In this study a considerable proportion of the isolates were identified as NTM. These were identified as slender pink rods upon ZN staining which is still used as the preliminary diagnostic tool in most of the developing countries mainly due to the cost effectiveness and easy performance [23]. The mycobacterial infections due to these organisms might present similar clinical symptoms mimicking TB, making the infection misdiagnosed as TB, hence therapy would include anti-tuberculous drugs. Precise identification of the *Mycobacterium* species before treatment can have a considerable influence on clinical management. Misdiagnosis of NTM bacilli as MTB and thereby considering it as pulmonary TB, can lead to unnecessary treatment of patients which could lead to the emergence of drug resistant *Mycobacterium* strains. Moreover, *Rhodococcus* spp which was identified is also an acid fast bacterium [24] which renders it difficult to discriminate from MTB and NTM. Therefore molecular identification methods are beneficial in the identification of such species prior to initiation of treatment.

There are incidences where *M. celatum* isolates had been isolated from bronchial washings [25] which was elucidated as a potential human pathogen [26]. *M. celatum* was first reported in 1993. This bacterium causes pulmonary infections similar to the infections caused by MTB and other NTM [19]. Even though, it was believed that this organism causes infection in patients who have depressed immunity there were reports describing pulmonary infections caused by *M. celatum* in immuno-competent patients [27]. The isolate from this study produced smooth, convex, non-pigmented colonies on L-J medium. Even though this species is known as a slow grower, the *M. celatum* isolate identified (KU179047) during this study was found to be rapidly growing bacterium which formed colonies on L-J medium in less than 7 days. The isolate was resistant to ciprofloxacin.

*M. avium*, *M. intracellulare*, *M. chelonae*, *M. abscessus* and *M. fortuitum* are the most common NTM pathogens found to be causing pulmonary infections [4]. Infections caused by MAC (*M. avium* and *M. intracellulare*) could lead to respiratory failure if untreated [28]. Increase in incidence of MAC was reported in early 1980’s simultaneously with the beginning of the AIDS widespread [29]. Though the two species, *M. avium* and *M. intracellulare* belong to a same group, in their pathogenicity and biology they show significant differences [30]. During this study three isolates were identified as MAC by multiplex Real-time PCR assay, but when sequenced, two isolates were confirmed as *M. intracellulare* and the other as *M. avium*. The *M. avium* isolate (KU198857) and one of the *M. intracellulare* isolates (KU195319) were resistant to the drug clarithromycin while the other *M.intracellulare* isolate (KU195323) was sensitive to all the tested drugs.

Infections caused by MCAG consisting of rapidly growing mycobacteria are a serious public health problem. MCAG consists of *M. immunogenum, M. massiliense, M. bolletii*, and *M. salmoniphilum* apart from *M. abscessus* and *M. chelonae* [10]. These species cannot be differentiated by biochemical testing and their 16S rRNA gene sequences are often similar, which renders it difficult to discriminate them individually. There were three MCAG isolates (KU179046, KU195328, and KU195330) identified during this study in which two isolates were resistant to ciprofloxacin while the other was resistant to both the clarithromycin and ciprofloxacin.

In most of the developing countries AFB positive smear microscopy is the tool to initiate anti TB treatment. However before the availability of the culture reports patients clinical conditions may deteriorate due to many factors such as side effects of anti TB therapy, drug resistance or if suffering from an infection due to NTM. Thus the results of this described molecular assay can help to expedite the decision making process so that relevant treatment could be initiated without delay.

## Conclusion

In conclusion, the optimized SYBR green mediated multiplex real time PCR procedure described, is rapid and simple to perform and could assist in identifying and differentiation of pathogenic *M. tuberculosis* complex from the opportunistic non tuberculous mycobacteria, which will allow early initiation of treatment depending on the causative agent.

## Abbreviations

AFB: Acid fast bacilli
CTAB: N-Cetyl-N, N, N-trimethyl ammonium bromide
INH: Isoniazid
ITs: Internal transcribed spacers
L-J medium: Löwenstein–Jensen medium
MAC: *M. avium* complex
MCAG: *M. chelonae-M.abscessus* group
MDR: Multi-drug resistant
MFG: *M. fortuitum* group
MTC: *Mycobacterium tuberculosis* complex
NTM: Non-tuberculous Mycobacteria
PCR: Polymerase chain reaction
PNB: P-nitrobenzioc acid
RIF: Rifampin
TB: Tuberculosis
TCH: Thiophene – 2 carboxylic acid hydrazide
ZN stain: Ziehl–Neelsen stain.
